# A Pavlovian account for paradoxical effects of motivation on controlling response vigour

**DOI:** 10.1038/s41598-019-43936-7

**Published:** 2019-05-20

**Authors:** Delphine Oudiette, Fabien Vinckier, Emmanuelle Bioud, Mathias Pessiglione

**Affiliations:** 1 Motivation, Brain and Behavior lab, Institut du Cerveau et de la Moelle épinière (ICM), Inserm U1127, CNRS U7225, Sorbonne Universités, Paris, France; 2 Service des Pathologies du Sommeil, Hôpital de la Pitié-Salpétrière, Assistance Publique Hôpitaux de Paris, Paris, France; 3 Département de Psychiatrie, Service Hospitalo-Universitaire, Centre Hospitalier Sainte-Anne, Paris, France

**Keywords:** Motivation, Motor control

## Abstract

In high stakes situations, people sometimes choke under pressure, performing below their abilities. Here, we suggest a novel mechanism to account for this paradoxical effect of motivation: the automatic adjustment of action vigour to potential reward. Although adaptive on average, this mechanism may impede fine motor control. Such detrimental effect was observed in three studies (n = 74 in total), using behavioural tasks where payoff depended on the precision of handgrip squeezing or golf putting. Participants produced more force for higher incentives, which aggravated their systematic overshooting of low-force targets. This reward bias was specific to action vigour, as reward did not alter action timing, direction or variability across trials. Although participants could report their reward bias, they somehow failed to limit their produced force. Such an automatic link between incentive and force level might correspond to a Pavlovian response that is counterproductive when action vigour is not instrumental for maximizing reward.

## Introduction

During a professional golf tournament, a famous champion once missed a hole that was only one meter away on the green. ‘Even I can do it,’ said someone in the audience. The champion held the club out to the amateur and challenged him: ‘you show us’. The amateur took up the challenge and … executed a perfect putt. ‘But this is not the real game,’ said the champion. ‘I want you to try again, and if you succeed, you can pocket this one-million-dollar check.’ The amateur tried, and missed.

As illustrated in this legendary anecdote, our skills sometimes fail us when we need them the most. This is a paradoxical effect of motivation because performance should scale with expected reward, if the behaviour were to follow on rational principles. Indeed, optimal control theory, which accounts well for behavioural performance in a variety of tasks, posits that control resources are allocated so as to minimize costs and maximize benefits^1–3^. More control should, therefore, be exerted when more money is at stake, leading to a better performance. More precisely, greater control should be exerted when the instrumentality of performance (i.e., in economic terms, its marginal utility) is higher, meaning when each unit of performance has a higher impact on the outcome^4^.

However, paradoxical effects of monetary incentives have been reported not only in sports but also in academic and business settings^5,6^. These paradoxical effects have been assimilated to a phenomenon known as ‘choking under pressure’, whose broad sense is performing sub-optimally when desire to perform well is maximal. After the seminal observation of an inverted U-shaped relationship between learning performance and the importance of potential outcomes^7^, choking has been interpreted as arising from hyper-arousal or over-motivation. These notions are compatible with two neuroimaging studies that have linked performance decrements for high incentives with activity in reward circuits (ventral striatum and midbrain area) and with individual measures of either financial motivation to gain money or loss aversion^8,9^. However, hyper-arousal and over-motivation remain quite vague as cognitive explanations of pressure effects.

Two specific cognitive theories were later proposed and extended to include the detrimental effect of any external stressor, typically the presence of an audience (for reviews see^10–14^). The distraction theory assumes that pressure directs attention towards task-irrelevant thoughts and worries, which reduces the amount of attentional control devoted to the task and thus degrades performance. The explicit monitoring theory assumes the opposite: pressure triggers excessive attentional control, which prevents the actor from using previously optimized procedural routines and thus disrupts performance. Both theories have received empirical support and are considered as valid explanations of pressure effects on various motor and cognitive skills, including golf putts, tennis serves, basketball free throws, working memory, mathematical problem solving, etc.^15–22^. However, they do not specify which aspect of motor or cognitive processes is disrupted under pressure.

Here, we suggest a more specific explanation for the detrimental effect of increasing incentives. Our idea is that potential rewards trigger direct specifications of behaviour, which may antagonize correct performance. This idea follows on the notion of Pavlovian behaviour, meaning an automatic response to a specific cue that bypasses a proper cost-benefit analysis of the situation^23–25^. Pavlovian responses are considered to be evolutionarily ancient and hard-wired in brain circuits. Even in species with a well-developed prefrontal cortex, such as primates, they can offer a good compromise between shortening deliberation time and adjusting behaviour to the context. Indeed, Pavlovian responses are supposed to be adapted on average, at least for the environment in which they were naturally selected. The persistence of Pavlovian responses is therefore key to understanding human behaviour and why it may be maladaptive in certain situations of the modern world.

Specifically, we hypothesized that incentives energize behaviour, in the sense that potential rewards boost action vigour, which can be measured as force in Newtons. This may not sound novel, as the link between expected reward and physical effort allocation is both intuitive and well supported by empirical studies (for recent reviews see^26–28^). What we add here is that the link is somewhat automatic: more physical effort may be allocated to action with higher expected reward, even when doing so is not instrumental (i.e. when it does not bring more reward). A Pavlovian account also makes a prediction about when pressure effects are adaptive: in situations where reward magnitude or probability increases with action vigour. One may speculate that this sort of situation was frequent in ancient evolutionary times – when, for instance, running faster would increase the probability of catching a prey.

In previous studies, we have explored this sort of adaptive situation using a task where monetary reward is proportional to handgrip force. Specifically, the proportion of the monetary incentives that participants win corresponds to the percentage of the maximal force that they produce. The standard behaviour in this task is to produce more force for higher incentives, with incentives being varied on a trial-by-trial basis (e.g.^29–31^). To test the situation where action vigour is not instrumental, we simply turned the force task into a precision task by changing the payoff rule: the eventual reward is now proportional to the distance between the produced force and a target force. Thus, payoff is orthogonal to action vigour: in order to maximize reward, force must not be too low or too high. An automatic link between incentive and force would even be detrimental in cases where targets tend to be overshot, and adaptive in the opposite case. The critical prediction is therefore that increasing incentives should aggravate the overshooting of low-force targets, leading to a paradoxical effect of motivation on controlling action vigour.

To be honest, we initially observed this link, hereafter termed ‘reward bias’, in two experiments that were designed for other purposes^32^ (Bioud *et al*., unpublished data), and which are presented here as supplementary information. In the following, we report three replication studies, where we first validate the concept of a reward bias, then isolate incentive effects on force from potential effects on timing and direction, and finally generalize the phenomenon to a more ecological paradigm (golf putting).

## Results

**Figure 1:**
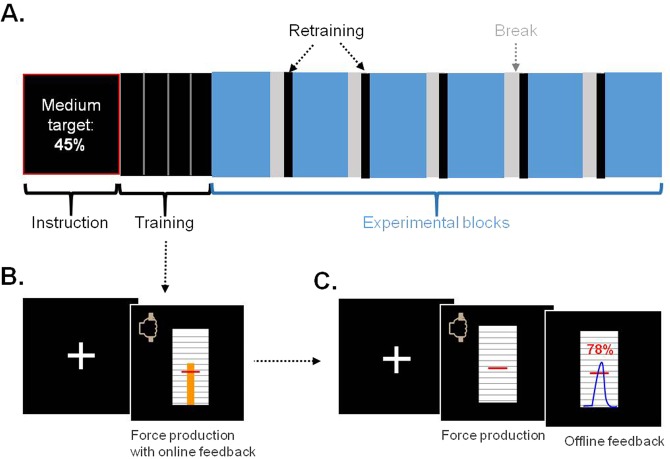
General design (all three experiments). (**A**) All experiments started with progressive training on the motor performance involved in the behavioural task. The actual experiment was divided into sessions, each corresponding to a specific target (only one session is represented here). Each session contained 6 blocks of 18 trials, separated by short breaks and retraining trials. An instruction screen at the beginning of each block indicated the next targets. In the different experiments, participants had to control force only (Exp 1), force and time (Exp 2), force and direction (Exp 3). (**B**) Illustration of a training trial with online feedback during force production (Exp 1). The target force is represented by the red line. The amplitude of the orange bar increased online proportionally to participants’ force level. The goal was to bring the top of the bar to the target force, with a single short pulse. (**C**) Illustration of a training trial without online feedback but with offline feedback (Exp 1). The goal was to produce a force pulse whose peak matched the target. The offline feedback shows the force profile across the trial and the performance, expressed as a percentage of the optimum (100% means exactly on target).

For all three experiments presented in the main text, participants were trained at a motor precision task (hand gripping or golf putting), first with online visual feedback, then with offline feedback only, and finally without any feedback on performance, as in the test blocks (see Fig. 1). The training trials enabled participants calibrating the motor command required to hit the target. In test trials, they only had proprioceptive feedback on their hand movement, but no visual feedback on the distance to target. Of note, when provided with a visual feedback, participants were on target at every trial, because they could correct their initial force pulse. Suppressing visual feedback was therefore crucial to get a non-trivial task where participants make errors. Between test blocks, they had a short retraining session with performance feedback again, to maintain calibration throughout the experiment.

Critically, participants’ degree of motivation was manipulated in test blocks by varying the amount of reward at stake (i.e., the maximal incentive) on a trial-by-trial basis (6 levels ranging from 5c to 50€). Participants were instructed that their payoff for each trial would be inversely proportional to the distance from target. Thus, they would win the full reward if they were exactly on target and nothing if they were at maximal distance (i.e., staying on start position or reaching a symmetrical position on the other side of target). In a subset of trials, they were also asked to position their shot with respect to target, in order to get a subjective estimation of their performance, and thereby assess their knowledge of potential bias.

### Experiment 1

**Figure 2:**
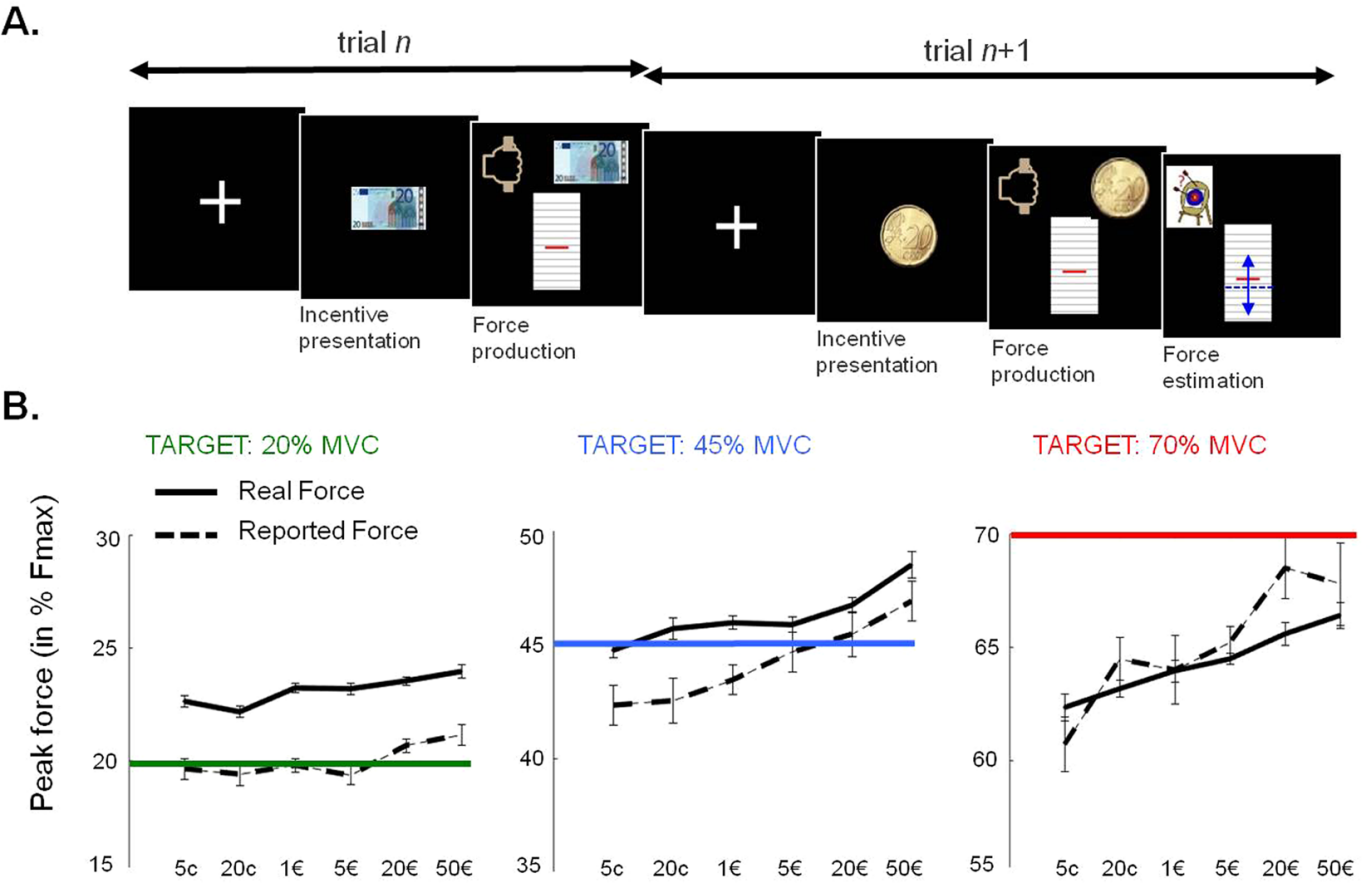
Influence of motivation on force precision (Exp 1). (**A**) Example trials. Participants were first presented with an image of the incentive (potential gain). Then a fist and a target image (red line) appeared to trigger force production. Participants had to squeeze a handgrip such that their peak force hit the target. The target was always at the same position on screen but could correspond to either 20, 45 or 70% of maximum voluntary contraction. In one third of trials, participants were asked to estimate their performance by positioning a cursor (in blue) on their perceived peak force, relative to the target. (**B**) Variations of peak force as a function of incentive level and target force. Participants tended to produce more force for higher incentive level, which aggravated the (underestimated) overshooting of low targets. Data points are group-level means ± inter-subject s.e.m. Variance between individual means has been removed from error bars to better illustrate the effect of incentives.

In experiment 1, 24 participants performed a motor precision task (Fig. 2A) which consisted in squeezing a handgrip such that peak force would be as close as possible to a given target force level that was either low (20% of MVC), medium (45% of MVC), or high (70% of MVC). Trial-by-trial series of peak forces were analyzed with a simple linear model that included a constant (to assess the offset of motor performance), the incentive level (to assess the effect of motivation) and the trial index (to assess the effect of fatigue). This model was fitted separately for the different target forces. The same analysis was applied to peak force estimates that participants provided in one third of trials.

#### The motor bias: participants overshot low target force and undershot high target force

To assess motor performance we compared the constant parameters (irrespective of incentive level and trial index) with target force level. Participants overshot low targets (22.7 ± 0.99 vs. 20%, t(23) = 2.7; p = 0.012) and undershot high targets (63.9 ± 1.4 vs. 70%, t(23) = −4.5, p = 0.0002). They were on target only for medium level (45.9 ± 0.94 vs. 45%, t(23) = 0.95; p = 0.35). Thus, the low and high targets seemed more difficult to hit, possibly because small and big forces are harder to produce (see Fig. 2B). To test this idea, we presented target forces two by two and asked participants which they would choose if they had to do one more session of testing: participants preferred low to medium targets (73 vs. 28%) medium to high targets (68 vs. 32%), and low to high targets (82 vs. 18%). Such preference pattern suggests that perceived cost was related to physical effort more than task difficulty.

#### The reward bias: participants squeezed harder for higher rewards, even for low targets

To assess motivation effects we compared the regression weight on incentive level to zero. For every target we found a significantly positive effect on force production (target = 20% MVC, β_Incentive_ = 0.52 ± 0.13, t(23) = 4.0, p = 0.0006; target = 45%, β_Incentive_ = 0.99 ± 0.27, t(23) = 3.7, p = 0.0012, target = 70%: β_Incentive_ = 1.4 ± 0.32, t(23) = 4.2, p = 0.003). Thus, the higher the prospective incentive, the harder participants squeezed the handgrip, even when it was detrimental to do so, such as when target force was low (see Fig. 2B). This reward bias was larger for high targets compared to low ones (high vs. low target, t(23) = 2.4, p = 0.022, high vs. medium target, t(23) = 0.86, p = 0.39; medium vs. low target, t(23) = 1.6, p = 0.13).

#### The decalibration: the motor bias was aggravated with time on task

To assess fatigue effects we compared the regression weight on trial index to zero. Trial index was initiated at the beginning of each block of 18 trials. Across trials (but within blocks), participants overshot more and more the low targets (target = 20%, β_Trial_ = 0.62 ± 0.21, t(23) = 3.0, p = 0.007) and undershot more and more the high targets (target = 70%, β_Trial_ = −1.4 ± 0.49, t(23) = −2.9, p = 0.0078). There was a non-significant trend for an increase in force over trials for medium targets (target = 45%, β_Trial_ = 0.79 ± 0.39, t(23) = 2.04, p = 0.054). Note that between blocks, participants were recalibrated with visual feedback allowing comparison of performance to target force. Thus, decalibration is not due to muscular fatigue, which would globally decrease force, but to mere forgetting. Indeed, in the absence of feedback, participants regressed to the middle of force range, as if the mapping between motor command and target force had to be learned again.

#### Subjective estimation: a reward bias but no overshoot

A similar linear regression on force estimates revealed that participants were aware of the reward bias: they reported higher force production for higher incentives, irrespective of target level (target = 20%, t(18) = 3.08, p = 0.0065; target = 45%, t(18) = 3.0, p = 0.0076; target = 70%, t(18) = 4.1, p = 0.0007). However, participants seemed not fully aware that they were overshooting low targets: as shown by the comparison of intercepts, estimated force was significantly below produced force (target = 20%, 19.9 ± 0.57% vs. 22.2 ± 0.98%, t(18) = −2.89; p = 0.0096). There was no significant difference between estimated and produced force for medium and high targets. Furthermore, force estimates were not significantly modulated by trial index, whatever the target force.

#### Control of variability: no effect of incentive motivation

In this precision task, motivation induced by potential reward may be expected to improve control of grip force, and therefore to decrease the variability of forces produced across trials. To test this possibility, we simply regressed inter-trial variance against incentive level. We found no significant effect (target = 20%, t(23) = 1.3, p = 0.19; target = 45%, t(23) = −1.5, p = 0.14; target = 70%, t(23) = −1.04, p = 0.31). This suggests that higher incentives increased the amount of force produced but did not reduce its variability, which could have mitigated the detrimental impact of the reward bias on performance.

### Experiment 2

**Figure 3:**
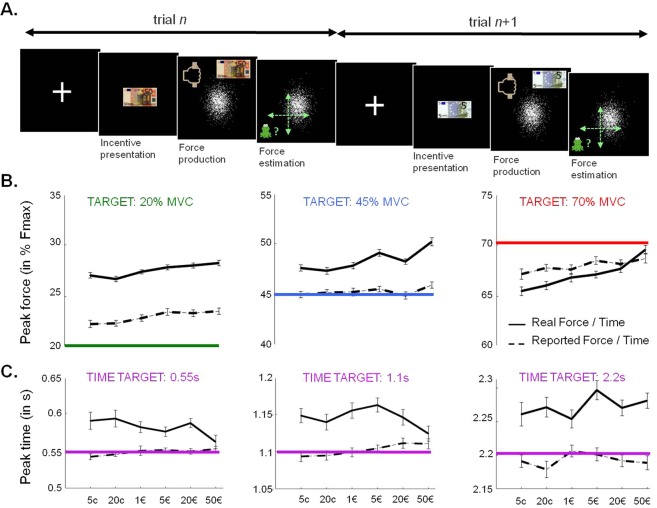
Influence of motivation on force and timing precision (Exp 2). (**A**) Example trials. Participants were first presented with an image of the incentive (potential gain). Then a fist and a cloud image (white points) appeared to trigger force production. The task worked as if a frog was passing from left to right at a constant speed and intended to hit the centre of the cloud to collect as many bugs as possible. Thus, participants had to squeeze a handgrip such that the peak force would hit the target force at the right moment (target time). The target (cloud centre) was always at the same position on screen but could correspond to different force and time targets. In all trials, participants were asked to estimate their performance by positioning a cursor (in green) on their perceived peak force, within a two-dimensional (time x force) space centred on the target. (**B**) Variations of peak force as a function of incentive level and target force. Participants tended to produce more force for higher incentive level, which aggravated the (underestimated) overshooting of low targets. The same target time (1.1 s) was used for all three target forces. (**C**) Variations of peak time as a function of incentive level and target time. Participants tended to overshoot target times, and to underestimate this overshoot, but there was no consistent, detrimental effect of incentive level on their timing. All three target times were tested with the medium target force (45% MVC). Data points are group-level means ± inter-subject s.e.m. Variance between individual means has been removed from error bars to better illustrate the effect of incentives.

In experiment 2, we tested for the specificity of the reward effect, by adding time as a second dimension that participants had to control. The question was whether the reward bias would also be reflected in response time, meaning that people would go not only stronger but also faster for bigger reward. Thus, 26 participants performed a variant of the motor precision task (Fig. 3A), in which they had to squeeze a handgrip such that the peak would not only hit a predefined target force level (20%, 45% or 70% of MVC as in experiment 1), but also do it at the right time (0.55 s, 1.1 s or 2.2 s after the go signal). Fast and slow targets (0.55 s and 2.2 s) were only tested with the medium target force (45% of MVC). So comparison of force targets was only done for the 1.1 s timing, and comparison of time targets for the 45% force. Peak force and time were analyzed separately for the different targets, as in experiment 1, with a simple linear model that included incentive level and trial index. The same analysis was applied to perceived peak force and time, which participants indicated in all trials.

#### The motor bias

Participants overshot not only low force targets (27.5 ± 1.4% vs. 20%, t(25) = 5.5; p < 0.0001) but also medium force targets (48.4 ± 1.6% vs. 45%, t(25) = 2.2, p = 0.039), and had a non-significant tendency to undershoot high force targets (67.1 ± 1.9% vs. 70%, t(25) = 1.2, p = 0.14). The overshoot of low targets was even larger than in the previous experiment (22.7 ± 0.99% in Exp 1 vs. 27.5 ± 1.4% in Exp2; t = −2.9; p = 0.0066), possibly because the demand for controlling a second dimension acted as a stressor or distractor. Regarding timing, participants were slightly slower than required for all time targets (fast target: 0.59 ± 0.007 s vs. 0.55 s, t(25) = 3.4, p = 0.0024; medium target: 1.15 ± 0.008 s vs. 1.1 s, t(25) = 2.7, p = 0.014; slow target: 2.27 ± 0.017 s vs. 2.2 s, t(25) = 2.9, p = 0.008).

#### The reward bias

Similarly to experiment 1, we found that higher rewards predicted higher force (see Fig. 3B), for every target (target = 20%, β_Incentive_ = 0.51 ± 0.14, t(25) = 3.7, p = 0.001; target = 45%, β_Incentive_ = 0.82 ± 0.14, t(25) = 6.1, p < 0.0001; target = 70%, β_Incentive_ = 1.2 ± 0.24, t(25) = 5.0, p < 0.0001). This reward bias was not observed in the time dimension, except for fast targets (target = 0.55 s, β_Incentive_ = −0.0084 ± 0.0011, t(25) = −2.3, p = 0.029; target = 1.1 s; β_Incentive_ = −0.0055 ± 0.0025, t(25) = −1.5, p = 0.15; target = 2.2 s, β_Incentive_ = 0.0074 ± 0.0037, t(25) = 1.3, p = 0.22). However, contrary to the reward bias observed in force level, the slight speeding effect observed with fast targets was not detrimental, as it led timing closer to that required.

#### Decalibration

As seen in experiment 1, the overshooting of low targets was aggravated with trials without feedback (target = 20%, β_Trial_ = 0.54 ± 0.21; t(25) = 2.6; p = 0.017), as was the undershooting of high targets (target = 70%, β_Trial_ = −1.80 ± 0.53; t(25) = −3.3, p = 0.0027). This corresponds to the decalibration effect already observed in experiment 1. The tendency to squeeze slower than required was even amplified with the number of trials for all targets (target = 0.55 s, β_Trial_ = 0.012 ± 0.0016, t(25) = 2.7; p = 0.013; target = 1.1 s, β_Trial_ = 0.026 ± 0.0023, t(25) = 4.2, p = 0.0002; target = 2.2 s, β_Trial_ = 0.04 ± 0.0041, t(25) = 4.1, p = 0.0004). Contrary to the decalibration effect observed on force level, this can be distinguished from a regression to the average target, and may arise from physical fatigue, which would slow down force production. Alternatively, the general slowing could mean that participants had a natural tendency to underestimate duration, which was partially corrected by training.

#### Subjective estimates

We found again a reward bias in force estimates but only for low and high targets (target = 20%, β_Incentive_ = 0.50 ± 0.18; t(25) = 2.8, p = 0.0092; target = 45%, β_Incentive_ = 0.2 ± 0.15, t(25) = 1.4, p = 0.19; target = 70%, β_Incentive_ = 0.49 ± 0.23, t(25) = 2.2, p = 0.039). As in experiment 1, reported force was lower than produced force and thus closer to the target, indicating that participants underestimated the overshoot of low target (target = 20%, 23.1 ± 0.93% vs. 27.5 ± 1.4%, t(25) = −3.2; p = 0.0037).

Similarly, time estimates were shorter than actual times, and closer to the targets (target = 0.55 s, 0.55 ± 0.0065 vs. 0.59 ± 0.0065 s, t(25) = −2.5, p = 0.018; target = 1.1 s, 1.10 ± 0.008 vs.1.15 ± 0.008, t(25) = −2.6, p = 0.014; target = 2.2 s, 2.20 ± 0.077 vs. 2.27 ± 0.017, t(25) = −2.6 p = 0.014).

#### Control of variability

Consistently with experiment 1, we found no significant effect of reward on force variability (target = 20%, t(25) = 0.76, p = 0.46; target = 45%, t(25) = 1.0, p = 0.33; target = 70%, t(25) = 0.44, p = 0.66), suggesting again that higher incentives increased the amount of force produced but not the quality of motor control. Similarly, incentive level had no significant effect on time variability (target = 0.55 s, t(25) = −1.5, p = 0.14; target = 1.1 s, t(25) = −0.47, p = 0.65; target = 2.2 s, t(25) = −1.7, p = 0.095).

### Experiment 3

In the first two experiments, we found that participants produced more force when potential reward was higher - an effect that was detrimental when small force was required (20% MVC target). However, this detrimental reward bias was linked to the overshoot of low targets, which could be due to the presence of higher targets in the same experiments. In other words, overshooting could arise from a regression to the mean target, because participants might forget about the mapping between proprioceptive input and the different required force levels, as shown with the decalibration effect. The first aim of experiment 3 was to probe reward bias and overshoot in a situation where only one single fixed target was presented during the entire experiment, to avoid any confusion that could happen when different target levels are presented within the same experiment. The second aim was to disentangle between the force dimension and the precision of movement, which were confounded in previous experiments. The idea was to test whether during more complex gesture, excessive force would translate in a general disorganization of movement control. The last aim was to assess whether our effects of interest would occur in a more ecological situation.

**Figure 4:**
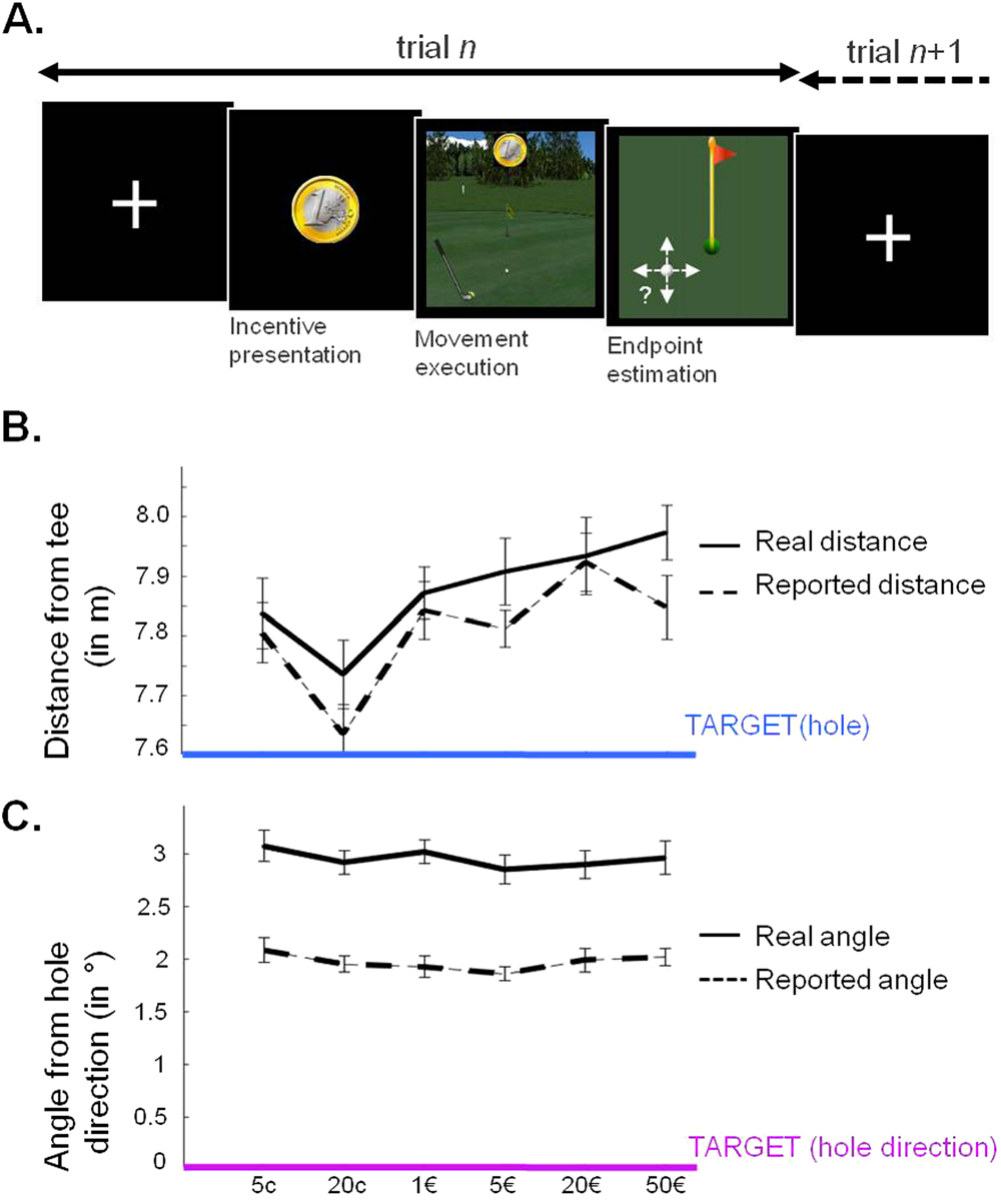
Influence of motivation on force and direction precision (Exp 3). (**A**) Example trials. Participants were first presented with an image of the incentive (potential gain). Then, a golf green appeared to trigger force production. Participants had to put a golf ball so as to hit the hole, which was always located at the same position. Thus, they had to control both their force, which determined the distance travelled by the ball (from the tee), and the direction of their movement, which determined the angle of ball trajectory (from the hole direction). (**B**) Variations of distance as a function of incentive level. Participants tended to produce more force for higher incentive level, which aggravated the (underestimated) overshooting of the hole. (**C**) Variations of (unsigned) angle as a function of incentive level. Participants underestimated their error in angle, but there was no effect of incentive level on the direction of their movement. Data points are group-level means ± inter-subject s.e.m. Variance between individual means has been removed from error bars to better illustrate the effect of incentives.

We therefore employed a golf simulator to examine putting movement, in another sample of 24 participants (see Fig. 4A). The distance to the hole was kept constant (7.6 m) and potential rewards were varied as in previous experiments. Our dependent variables were the distance travelled by the ball (an indirect measure of the force exerted on the ball with the club) and the (unsigned) angle deviation from the straight line to the hole (a measure of direction accuracy). In all trials, participants also had to estimate the endpoint of their ball trajectory, with respect to the hole. This estimate was decomposed into perceived distance and angle. As before, actual force and direction were analyzed separately with a linear model including incentive level and trial number. The same linear model was fitted to perceived force and direction.

#### The motor bias

Just like with the low target force in the first two experiments, the model intercept showed that participants tend to overshoot the hole on average, independently of incentive level, but this effect was not significant (7.9 ± 0.19 vs. 7.6 m, t(23) = 1.6, p = 0.13, see Fig. 4B).

#### The reward bias

Consistently with the first two experiments, higher force was exerted for higher incentive level, such that a longer distance was covered by the ball (β_Incentive_ = 0.07, t(23) = 2.6, p = 0.016). Because we noticed a slight non-linearity, we tested the possibility of a quadratic relationship between distance and incentive level, by squaring the (z-scored) incentive regressor. The quadratic effect of incentive level was not significant (t(23) = 0.23, p = 0.82), discarding an inverted U-shaped relationship between performance and incentive level. Moreover, when we included both the linear and quadratic functions of incentive level in the same GLM, the linear link was still significant (t = 2.6, p = 0.018), but the quadratic link was not (t = 0.29, p = 0.18). In contrast to the effect on distance, incentive level had no significant influence on direction accuracy, i.e. the unsigned angle between ball trajectory and straight line (GLM, β_Incentive_ = −0.029, t(23) = −0.47, p = 0.64).

#### Decalibration

The overshoot was aggravated with time on task: participants sent the ball further away from the hole as the number of trials without feedback increases (β_Trial_ = 0.18, t(23) = 3.4, p = 0.0024). Contrary to previous experiments, this decalibration of force production cannot be interpreted as a regression to mean target; it may rather represent a natural tendency to exert too much force, which must be tempered by training. In contrast to force, there was no effect of trial number on direction accuracy (GLM, β_Trial_ = 0.03, t(23) = 0.54, p = 0.59).

#### Subjective estimates

As in previous experiments, there was a trend for the reward bias to be reflected in subjective estimates of distance, but this trend was not significant (β_Incentive_ = 0.05; t(23) = 1.6, p = 0.12). Participants also tended to underestimate the overshoot, but perceived distance was not significantly shorter than the actual distance reached by the ball (7.8 ± 0.15 vs. 7.9 ± 0.18, t = −0.47, p = 0.64). However, participants significantly underestimated the deviation from target direction, as shown by angle estimates (1.97 ± 0.10 vs. 2.95 ± 0.16°; t(23) = −11.2; p < 0.0001).

#### Control of variability

Contrary to previous experiments, we observed a significant decrease in force variability for higher incentive level (t(23) = −2.58, p = 0.017). This suggests that motivation helped participants to exert better control on their force, without correcting for their reward bias. There was no effect of incentive level, however, on angle variability (t(23) = −1.41, p = 0.17).

In this result section, we have provided statistical evidence in favour of the reward bias separately for each experiment. Note that each of the three independent replications would hold if we applied a correction for multiple comparisons to the significance threshold. Our analysis is more conservative than pooling the experiments and calculating a global reward bias. Indeed, the global p-value of the β_Incentive_ estimates over the three main experiments (n = 74 participants) was p = 2.91*10^−10^.

### Supplementary experiments

We provide as supplementary material some key information about the experimental design and results of the two experiments in which we first observed the reward bias. These experiments were performed for other purposes, as explained in corresponding papers, before the preceding main three experiments. However, they both assessed the reward bias, because they involved producing low forces for varying incentive levels. Thus, they provide additional evidence in other groups of participants (n = 80 in total), using different versions of the experimental design. They bring valuable information about the generality of the reward bias because 1) they use a single (low force) target, as in the golf experiment, thus avoiding any possibility of confusion between targets, 2) they include an additional factor with potential loss (in case of poor performance), which did not exert any bias on force production and 3) they implemented a binary payoff procedure (gain/loss depending on hitting/missing target) instead of the proportional rule (the closer to the target, the higher the payoff). More details can be found in supplementary figure legends.

## Discussion

Our results provide evidence for a reward bias, defined as the production of stronger force for higher incentive, even when this response is not adaptive. We initially observed this phenomenon in two studies tackling other issues^32^ (Bioud *et al*., unpublished data). We replicated this finding in three other studies, first with handgrip force precision and then with golf putting. Critically, the reward bias was detrimental in situations where small to moderate force had to be precisely exerted, because it aggravated the overshoot and thus increased the distance from target.

We believe this phenomenon is different from what has been subsumed under the ‘choking under pressure’ umbrella and traditionally explained by either distraction or explicit monitoring theories^11,12,14^. The effects were quite specific in our studies: the impact of incentives was restricted to action vigour, without affecting the timing or direction of movements. By timing we mean adjusting to target time the moment of reaching force peak. This can be done independently of action vigour, as can be the direction of movement to target location in 3D space. We note that the global null result on timing seems inconsistent with the intuitive notion that rewards speed behaviour, as we occasionally observed (with fast targets). The discrepancy likely comes from the fact that, in our design, reward was conditioned on timing precision, thereby penalizing both early and late responses. Also, the variability of performance was not aggravated by higher incentives, as either the relaxation of attentional control or trembling due to increased muscular tension would predict. Finally, we observed a linear relationship between peak force and incentive level, which does not match the theoretical inverted U-shaped function linking performance to arousal^7,33^. Importantly, when we included a quadratic function of incentive level in the model used to fit the data, the linear term remained significant, but the quadratic term was not. This confirms that force production was a linear function of incentive values. Note that we used ordinal values in our linear model. With cardinal values the function would become concave, thus contradicting the hyper-arousal theory, which would predict a convex function if choking dramatically exploded with increasing incentives. It is unlikely that we solely sampled the right half of a hidden U-shaped function, because our low incentive level (5 cents) was already quite negligible. Of course, our observations do not invalidate the other theories mentioned above. Rather, we see the reward bias as yet another pathway for pressure effects, which are likely to reflect a collection of various cognitive processes.

Our reward bias is better described as the activation of an automatic link between expected reward and action vigour. Indeed, participants noted that their force increased with incentive level but did not correct for this bias, as if it had been irrepressibly activated. This automatic impulse is therefore different from the subconscious motivation of effort exertion that has been obtained using subliminal presentation of potential rewards^30,34–37^. It remains possible that, even if participants reported having produced more force in high-incentive trials, they did not fully realize the systematic mapping from incentive level to force production. In the terminology of behavioural ecology^23–25^, the reward bias would thus be interpreted as the intervention of a Pavlovian controller, which would distort the action driven by the other controllers – the habit and goal-directed systems. This is at odds with the explicit monitoring theory, which could be interpreted in this framework as the intervention of a goal-directed controller, to the detriment of a better-suited habit-based performance. However, relative to habit-based, goal-directed control would only be detrimental in skilled participants, such as professional golfers in our last experiment.

At the neural level, the reward bias may correspond to the activation of the mesolimbic dopaminergic pathway including midbrain dopaminergic nuclei and the ventral striato-pallidal complex. Neuroimaging studies have implicated this pathway in mediating the effects of incentives on mental and physical effort, in situations where effort exertion is instrumental^30,38^. The link between incentive and effort is impaired by the disruption of this pathway, through lesions of the striato-pallidum complex^31^, and improved by the use of dopaminergic medication^29,39^. The ventral striatum has also been implicated in Pavlovian-to-Instrumental Transfer^40,41^, which supports its participation in both automatic and deliberate responses to potential rewards. The involvement of the mesolimbic dopaminergic pathway is also supported by a neuroimaging study relating ventral midbrain activity to performance decrement in a task where participants had to catch a prey in a computerized maze^9^. Moreover, this link was modulated by individual measures of motivation for money, which fits with the general idea that pressure effects arise from excessive motivation triggered by monetary incentives.

Another neuroimaging study^8^ found, on the contrary, that detrimental pressure effects on a motor control task were associated with a decrease in ventral striatum activity for the highest incentives. This association was modulated by individual measures of loss aversion, which leads to the interpretation that performance impairment was induced by the fear of losing potential rewards. This sort of interpretation would be compatible with distraction theory, although the link between being distracted by loss prospect and failing to control hand trajectory remains to be specified. We believe that this interpretation is less likely with regard to the reward bias, for two reasons. First, fear of losing is more salient in tasks where the outcome is binary (as when one has to hit a target), whereas the reward bias was observed here with both binary and proportional outcomes. Second, the positive link between incentive and force level was stronger when incentives were presented as potential gains versus potential losses (in supplementary studies). This dissociation distinguishes our pressure effect from ‘hyper-arousal’ interpretations, which would equally concern both the gain and loss domains, and justifies the use of the ‘reward bias’ label. The reward bias may reflect a natural pathway that makes invigoration of behaviour easier to trigger from potential reward than from potential punishment^42,43^.

Qualifying the reward bias as a Pavlovian response raises the question of why it has been naturally selected, or in other words, how it could enhance adaptive fitness. At first glance, the reward bias seems maladaptive because it may result in exerting more effort for obtaining less reward. Yet our results provide a potential explanation: this response is adapted to high target forces. It could be that a natural correlation exists between reward and effort magnitudes, meaning that on average, larger rewards require more effort to obtain them. The reward bias may thus reflect a prior that the brain could have formed through sampling, given these statistical contingencies. Another possibility is that the negative consequences of overshooting are negligible compared to those of undershooting. To take trivial examples: when chasing a prey, there is no harm in running too fast, or throwing a projectile with too much force (provided the direction is accurate). Thus, exerting too much force may be a mechanism that increases the probability of success, or reduces the delay of reward delivery, when there is some uncertainty in the action-outcome mapping. It implies that the benefit of increasing success likelihood would overcome the cost of exerting more effort. This would be an explanation not only for the reward bias (it is even more important to secure the outcome when it is more rewarding), but also for the motor bias (the mere fact of overshooting).

Indeed, beyond the reward bias, we also observed a motor bias: irrespective of incentive level, participants overshot small to moderate target forces. It is surprising that participants did not correct for this bias and ended up missing a substantial portion of potential rewards. A first explanation is that participants progressively forgot about the different individual targets, which we call the decalibration effect, and regressed to the mean of the target range. However, this would not explain the motor bias that we observed in experiments where there was only one target (golf putting and supplementary studies). A variant of this explanation is that participants were driven to overshoot low targets, and undershoot high targets, because we presented the target at the middle of the screen. This is unlikely because again, the motor bias was also observed when using a single target force, precluding the possibility of any confusion between targets. A second explanation is that participants regressed to an idiosyncratic default force, which would be, on average, around the medium target level (half their maximal force). One reason for having such a default could be that moderate forces are more frequent in everyday life and therefore set priors on force production. It is also possible that moderate forces are in fact less costly, either because exerting small forces require more control or because they require co-contractions and therefore the activation of more muscular fibers^44,45^. To test this idea we asked participants which target they would choose for an extra-session of the experiment. A majority favoured the low target, ruling out the idea that medium targets represent low-cost default forces. Even if they required less effort, it is surprising that participants preferred small targets, for which their performance was poorer on average, compared to medium targets. This result hints to a simpler explanation: participants were not aware of their motor bias, as indicated by their subjective estimates. The minimization of the motor bias in subjective report could arise from the desire to be on target, and thus relate to another cognitive bias such as overconfidence, wishful thinking or social conformism^46^. It could also be favoured by a difficulty in sensing the force through proprioception, in the absence of visual feedback. The implicit motor bias observed here is reminiscent of the finding during tit-for-tat exchanges that produced force is under-estimated compared to received force, which might account for force escalation in social conflict^47^.

Although obtained in a specific motor control task, these results may generalize to everyday-life situations, in sport and beyond, and to other types of pressure. For instance, the phenomenon of social facilitation^48^, meaning the improvement of performance induced by the presence of an audience, may be restricted to action vigour. Indeed, the presence of an audience was shown to increase grip force^49^, the effect being instrumental in this case, to counteract force decay. Previous studies showed that competitive contexts with an audience deteriorated the performance of pianists, on both the technical and artistic levels, because they stroked the keys with excessive force^50,51^. One could speculate that higher stakes may also undesirably boost the vigour of critical gestures made by professionals such as surgeons or dentists. However, several limitations may reduce the generalizability of our findings. First, the reward bias was observed in the absence of visual feedback – it would be easily correctable if visual information was available to adjust motor commands. Second, it affected a motor skill that was newly learned – it is possible that more habitual movements (requiring less motor learning), or the same movements performed by well-trained professionals such as golf champions, would remain immune to this reward bias (despite anecdotes claiming the opposite). Third, it may be dependent on individual traits such as impulsivity, which can be interpreted as a boost in action vigour with the aim of an earlier reward, or anxiety, which was shown to modulate the relationship between performance and potential outcomes^13^. Thus, the impact of our cognitive bias in other pressure effects seen in sport, academic and business settings remains to be explored.

In conclusion, we provide evidence for an automatic cognitive process that adjusts action vigour to potential reward, inducing performance decrements in motor control tasks. This reward bias does not impair control precision *per se*, as measured by the variability of motor outputs, but aggravates the systematic tendency to overshoot low targets. Numerous previous studies have reported qualitatively similar, but quantitatively more pronounced, effects of incentives in settings where action vigour was instrumental in maximizing reward (e.g.^29–31^). Taken together, these results suggest that incentive motivation is beneficial in situations when reward increases with action vigour (such as running and weightlifting), and detrimental when reward increases with action precision (such as golf putting or basketball free throws).

## Methods

### Subjects

The study was approved by the Salpêtrière Hospital Ethics Committee and carried out in accordance with the French guidelines and regulations. A total of 74 healthy participants were recruited (n = 24, 26, and 24 subjects in experiments 1, 2, and 3, respectively). They were screened out for exclusion criteria: history of neurological or psychiatric disorder and recent use of psychoactive drug. They were paid 15€ per hour plus an additional bonus depending on their performance at the task (see below). All subjects gave informed consent before taking part in the study.

### General design

In all three experiments, we measured how force exertion varied, on a trial-by-trial basis, as a function of the reward at stake in motor precision tasks. We used six levels of incentives that covered a large range of potential values (5c, 20c, 1€, 5€, 20€ and 50€), the latter being quite high compared to the standard payoff for participating in an experiment. Participants were instructed that their monetary bonus for a given trial would be proportional to both their performance and to the reward at stake. For example, a performance of 80% for a prospect reward of 20€ yielded a 16€ bonus (80% of 20€). They were also told that one bonus trial would be randomly drawn at the end of each session and that the final bonus would be calculated as the average of the bonus trials (making a total of 1 to 5 bonus trials, depending on the experiment, with a maximal bonus of 50€).

The three experiments followed the same structure (see Fig. 1). Participants first benefited from extensive training on the different targets, progressively suppressing feedback information. Then they performed behavioural test sessions, each comprising 6 blocks of 18 trials, with every incentive level presented 3 times in a block, following a pseudo-random order. To avoid learning confounds, no feedback was provided on motor precision during the actual experiment. Between blocks, participants benefited from a brief re-training (n = 5 trials with offline feedback) to ensure that they did not drift away from the target. At the end of a session, participants had a 5-min break before starting a new session.

### Behavioural tasks

In all experiments, stimuli presentation was programmed with Matlab Psychophysics Toolbox and projected on a PC screen (experiments 1 and 2) or a projector screen (experiment 3). Experiments 1 and 2 employed a power grip (Hand Dynamometer, Vernier Software and Technology, Beaverton, Oregon, USA) interfaced with Matlab so as to readout force in Newtons.

### Experiment 1

In experiment 1, participants (n = 24; mean age: 24.6 ± 3.2 y, 12 females) were required to squeeze a handgrip with a short pulse such that their peak force would be as close as possible to a target force. Before the actual experiment, participants were familiarized with the handgrip device. To estimate maximum voluntary contraction (MVC), each participant was requested to exert maximal force on the grip three times in a row (only the highest value was retained). MVC served as a reference to normalize the targets to individual musculature of participants.

During the experiment, the target was represented by a horizontal bar that was drawn on a vertical graduated scale that indicated force from 0 to MVC. The bar was always positioned at the middle of the screen, but the level of force required to reach it varied across three sessions. The three target forces were 20%, 45% and 70% of MVC and their order across sessions was counterbalanced between subjects. All the sessions were performed during the same day, with a few minutes break between sessions. Performance was calculated as:

$$Perf=\frac{Dmax-D}{Dmax}\ast 100,$$

with D being the distance between produced and required peak force and Dmax the maximal distance allowed (set to 20% of MVC). Thus, a perfect performance corresponding to peak force being exactly on target was scored 100%.

Three training phases allowed participants to learn producing the required target force. In the first phase (n = 6 trials), online visual feedback on exerted force was displayed as a cursor moving up and down within the scale. When the grip was released, an offline visual feedback was displayed, in the form of force profile over time. Participants could then learn from the distance between their peak force and the target force, which was shown graphically and expressed as percentage of optimal performance (see formula above). In the second phase (n = 10 trials), online visual feedback was suppressed so that participants could only use proprioceptive feedback to control their force. Offline feedback was still provided at the end of squeezing movement, in the same way as during first phase, so that participants had a chance to adjust their force on the next trial. In the third phase (n = 6 trials), participants had neither online or offline feedback, and therefore could not use any visual information to regulate their movement.

In the last phase of training (n = 12 trials), participants were exactly in the same conditions as in the actual experiment. They were first presented with a monetary stimulus (a picture of a coin or a bill) indicating the reward at stake for the trial. The stimulus was displayed for around 3.5 s, with a jitter. Then the scale and the target bar appeared, together with a hand image that served as a trigger for action, meaning that participants had to squeeze the handgrip and try hitting the target force, without any online or offline feedback. From time to time (every three trials on average), an image with arrows and question mark were displayed, prompting participants to estimate the force they just produced. To do so, they had to move a blue cursor with the up and down arrows on the keyboard to indicate where they thought their peak force was, relative to the target (red bar). The initial position of the blue cursor on the scale was drawn randomly from uniform distribution. No feedback on estimation accuracy was provided, but participants were informed that accurate estimations would lead to an additional monetary bonus. This additional bonus was simply proportional to estimation accuracy, with a maximum of 5€ corresponding to perfect judgment. A fixation cross appeared for 1.2 s before the start of the next trial. At the end of this training phase, the average performance over the 12 trials was presented.

Each block of the actual experiment was identical to this last training phase (except that they were 18 trials instead of 12). The 6 blocks of a session used the same target force, and participants were trained again before the next session to familiarize with the new target force.

At the end of the experiment, we asked participants to choose which target force they would prefer if they had to do one more session. The three possible choices (20% vs. 45%, 45% vs. 70% and 20% vs. 70% MVC) were presented twice, with the left and right options counter-balanced between repetitions. Choice was self-paced and participants indicated their preference by pressing left or right arrow of the keyboard.

Of note, only 19/24 subjects were included in the analysis of subjective estimates because the remaining 5 subjects did not follow the instructions (they always left the cursor on its initial position).

### Experiment 2

In experiment 2, participants (n=26, mean age: 22.8 ± 3.6 y, 15 females) were required to squeeze a handgrip, not only with a specific peak force but also a precise timing. Their goal was presented as to control a hungry frog and collect as many bugs as possible. The frog moved from left to right at a constant speed, and participants had to squeeze the handgrip at the right time and the appropriate force for its tongue to catch the heart of a bugs’ cloud (located exactly at the centre of the screen). The bugs were represented by white dots, generated from a Gaussian distribution around the centre of the screen, which was emphasized by a bigger white filled circle. Thus, the peak force and latency determined the vertical and horizontal position of the tongue, respectively, and therefore the number of bugs collected, which was translated into performance and rewarded with money. The required force level and timing varied across 5 sessions, such that three force levels (20, 45 and 70% MVC) were sampled for the medium latency (1.1 s) and three force latencies (0.55, 1.1 and 2.2 s) for the medium level (45% MVC). Session order was counterbalanced across participants, and all the sessions were performed during the same day, with a few minutes break between sessions.

Performance was inversely proportional to the two-dimensional distance of peak force and latency from the target (screen centre):

$$Perf=\frac{{D}{\max }\,-{D}}{{D}{\max }}\ast 100$$

With $D=\surd (force{D}^{2}+latency{D}^{2})$ and Dmax the distance from the screen corner to the centre (corresponding to worst possible performance).

Training followed the same steps as in experiment 1. After measurement of MVC, which was used to normalize force levels, participants performed 10 trials to familiarize themselves with the design. In this familiarization phase, the frog moved at the bottom of the screen from left to right, and participants understood that handgrip squeeze intensity and timing determined performance on the vertical and horizontal axis, respectively. Then they were trained on the specific force level and latency imposed in the upcoming test session, first with both online and offline feedback, then offline feedback only, and last without any feedback. After the familiarization phase, the frog did not appear anymore. A hand appeared on the top left corner along with a bugs’ cloud at the centre, indicating that the (invisible) frog started to move and that participants should squeeze the handgrip at the appropriate level and timing. Online feedback was a visual trace of time and force on the two-dimensional space materialized by the screen. Offline feedback consisted in a force profile over time (as in experiment 1), with a line showing the distance from peak to target, and performance expressed as percentage of maximum (being exactly on target).

During the final training phase, participants were also trained to estimate their own performance, by positioning a cursor on the screen with the computer mouse to indicate peak force. Again the initial position of the cursor was randomly selected from uniform distribution. Feedback on estimation accuracy was provided by showing the position of the actual peak force such that the distance from the reported peak force could be appreciated. Estimation accuracy was calculated as the two-dimensional distance between actual and reported peak force, in a same manner as performance (in percentage of maximum), and was also indicated on screen. Before starting the test session, participants performed a short block of trials identical to the actual experiment.

During the actual experiment, participants were first presented with the incentive (coin or bill picture), and then squeezed the handgrip without feedback. They were told that the incentive would be multiplied by their performance to calculate their payoff, as in experiment 1. In every trial, they also had to estimate their performance by positioning their peak force on screen. There was no feedback on performance estimation until the end of the experiment, but participants were aware that they could win an additional bonus up to 5€ as a function of estimation accuracy.

### Experiment 3

In experiment 3, participants (n = 24, mean age: 25.5 ± 3.9 y, 18 females) had to perform golf putts, with the distance to the hole fixed at 7.6 m. The goal was to send the ball as close as possible to the hole. To make the situation more ecological, we used an indoor golf simulator set-up (P3ProSwing, Sports Vision Technologies, Bethel, ME). A picture of a green course was projected on a giant screen in front of participants, who were standing on a mat where the ball starting point was positioned. The ball was surrounded with movement sensors that detected an infrared reflective reference placed on the club. Every trial, participants hit a real golf ball, which was stopped by a pillow placed along the wall, below the screen. The kinematic parameters of the club trajectory were recorded by the golf simulator software, and served to calculate the virtual endpoint of the ball, relative to the hole.

As in experiment 2, performance was based on the two-dimensional distance D between the hole and the ball endpoint, in the space observed from above. Technically, it was calculated using the angle of the trajectory (from the straight line) and the distance (from the starting line) that were recorded by the golf simulator. Performance was expressed in percentage, such that 100 means on target and 0 as far as the starting point (7.6 m):

$$Perf=\frac{Dmax-D}{Dmax}\ast 100$$

With $D=\surd (x{D}^{2}+y{D}^{2})$, yD being measured along the straight line (yD = 7.6 – distance) and xD the distance along the perpendicular axis, (xD = distance * tang (angle)).

After a quick familiarization phase, participants followed progressive steps as in previous experiments: first with offline feedback, then without any feedback and finally in the exact same conditions as during the actual experiment. Offline feedback consisted in an animation replaying the ball trajectory from starting to end point. At the end of the animation, the distance to the hole was indicated, with xD and yD on horizontal and vertical axes. Before the actual experiment, participants were also trained to estimate the ball endpoint. As in experiment 2, they first used the computer mouse to place the randomly positioned cursor (an image of the ball) on the two-dimensional space viewed from above and centred on the hole. Then they were shown the animation with the actual trajectory followed by the ball.

Incentives were introduced in the last phase of training as in the actual experiment. Participants were first presented with the incentive alone then they had to press a key for the golf course to appear so that they can perform their putt (at their own pace). When they were done, they had to pick up the ball, place it on start position and press another key to access the next screen, corresponding to the estimation phase. As in previous experiments, there was no visual feedback on performance or estimation, and participants were aware that they could win an additional bonus up to 5€ depending upon estimation accuracy.

### Statistical information

The main dependent variables were peak of produced force (expressed as percentage of MVC) in experiments 1 and 2, peak latency (time between ‘go signal’ and peak force, expressed in seconds) in experiment 2, distance from the tee (in meters) and unsigned angle between ball and hole direction (in degrees) in experiment 3. In addition, we collected subjective estimates of all these variables, expressed in the same units.

All variables were analyzed with the same general linear model (GLM), which contained a constant and two parametric z-scored regressors: incentive level (ordinal, not cardinal value) and trial number (within a block, i.e. before retraining). Thus for all dependent variables GLM were written as follows: DV = β_0_ + β_Incentive_*Incentive + β_Trial_*Trial. Regression estimates were meant to assess our main effects of interest: β_0_ for the motor bias (overshoot), β_Incentive_ for the reward bias and β_trial_ for the decalibration. Regression coefficients were estimated at the individual level, separately for the different targets, using the glmfit function in Matlab Statistical Toolbox (MATLAB R2015b; MathWorks). They were then tested using two-tailed paired t-test at the group level, against target for the motor bias and against 0 for the reward bias and decalibration effect. All results are reported as mean ± inter-subject s.e.m.

In addition, to assess precision control, we computed for every subject the variance of DV across trials, for incentive level. We then regressed this measure against incentive level at the individual level and tested regression estimates at the group level, using two-tailed t-test (against 0).

## Supplementary information

Supplemental Data

## Data Availability

The datasets analyzed during the current study are available from the corresponding author on reasonable request.
