# Supplementary material for: A Pavlovian account for paradoxical effects of motivation on controlling response vigour
Source: Sci Rep. 2019 May 20;9:7607. doi: 10.1038/s41598-019-43936-7 (PMC6527680; doi:10.1038/s41598-019-43936-7)
Supplement: Supplementary file 1 — Supplemental Data [file 41598_2019_43936_MOESM1_ESM.docx]

**A Pavlovian account for paradoxical effects of motivation on controlling response vigour**

Delphine Oudiette^1,2*^, Fabien Vinckier^1,3^, Emmanuelle Bioud^1^, Mathias Pessiglione^1*^

^1^ Motivation, Brain and Behavior lab, Institut du Cerveau et de la Moelle épinière (ICM); Inserm U1127 ; CNRS U7225 ; Sorbonne Universités, Paris, France

^2^ Unité des Pathologies du Sommeil, Hôpital de la Pitié-Salpétrière, Assistance Publique - Hôpitaux de Paris, France

^3^ Département de Psychiatrie, Service Hospitalo-Universitaire, Centre Hospitalier Sainte-Anne

^*^ **Corresponding authors**: [delphine.oudiette@gmail.com](mailto:delphine.oudiette@gmail.com), mathias.pessiglione@gmail.com

**Supplementary Figure legends**

**
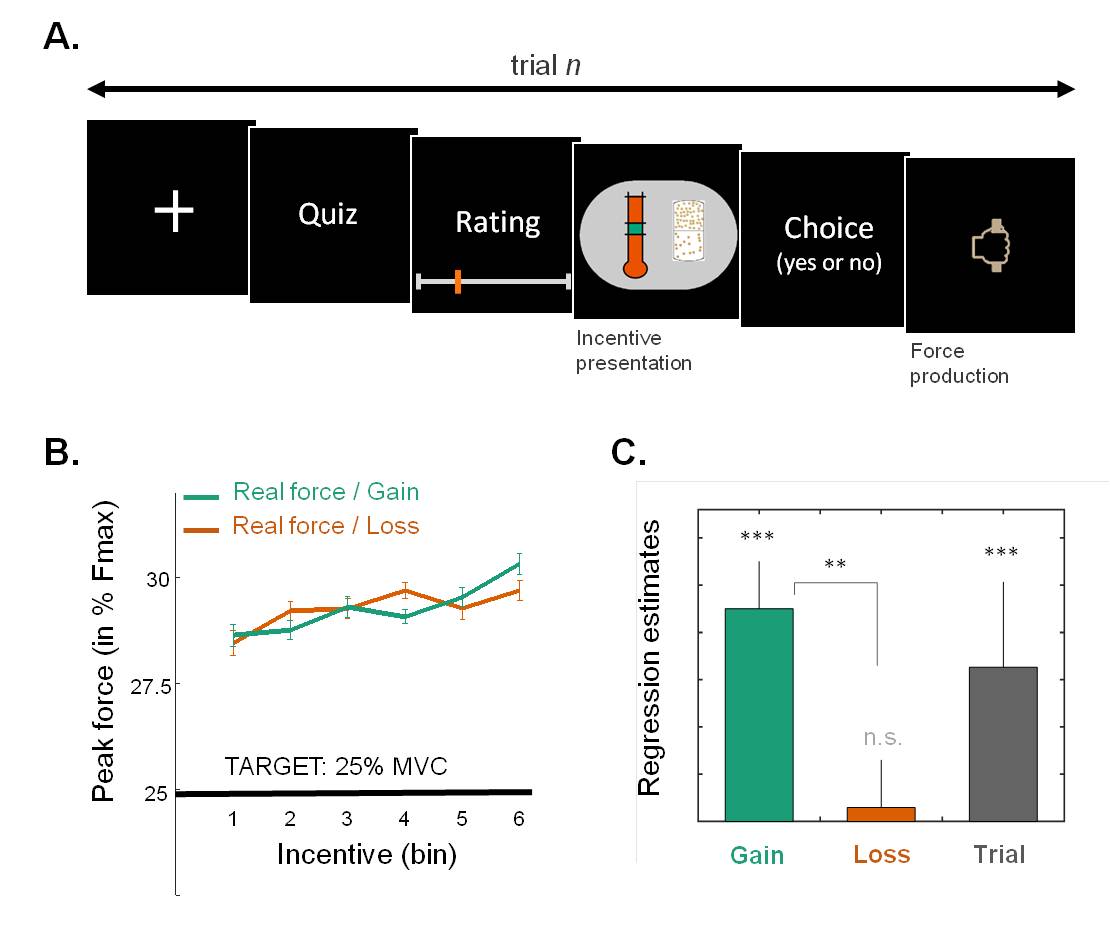
**

**Sup Figure 1. Influence of gain and loss prospects on force precision (Exp 4)**

Details about task design can be found in Vinckier et *al*., 2018 ^31^.

1. Example trial. Participants (n=61) performed two unrelated but interleaved tasks. The first was a cultural quiz, irrelevant to the present topic. The second involved both choice and force production, and can be considered as a variant of the motor precision tasks presented in the main text. Critical differences were that 1) due to the presence of a quiz task, and a rest period between the two tasks, force exertion was less frequent (one trial every 30 s on average), 2) the force target was always low (25% of maximal force), 3) the outcome was binary, depending upon whether force peak was within a force window around the target, 4) task difficulty (size of target window) was manipulated in addition to potential gain in case of success (10-cent coin images) and potential loss in case of failure (crossed coins), 5) participants could accept or decline the motor challenge, 6) there was no subjective estimation of force produced.
2. Variations of peak force as a function of both gain and loss levels. Participants tended to overshoot the target, and even more with higher incentive levels. Data points are group-level means ± inter-subject s.e.m. Variance between individual means has been removed from error bars to better illustrate the effect of incentives.
3. Regression estimates (betas) of a linear model fitted to trial-by-trial series of force peaks. The model included a constant, potential gain, potential loss, target size and trial index. Participants overshot the target (29.3±0.69 vs. 25%, t(60)=6.1; p<0.001). A significant positive modulation of peak force was found for potential gain, target size and trial index (all p<0.001), but not for potential loss (t(60)=0.3; p=0.77). Moreover, the direct comparison of reward vs. loss estimates was significant (t(60)=3.0; p=0.003).

**
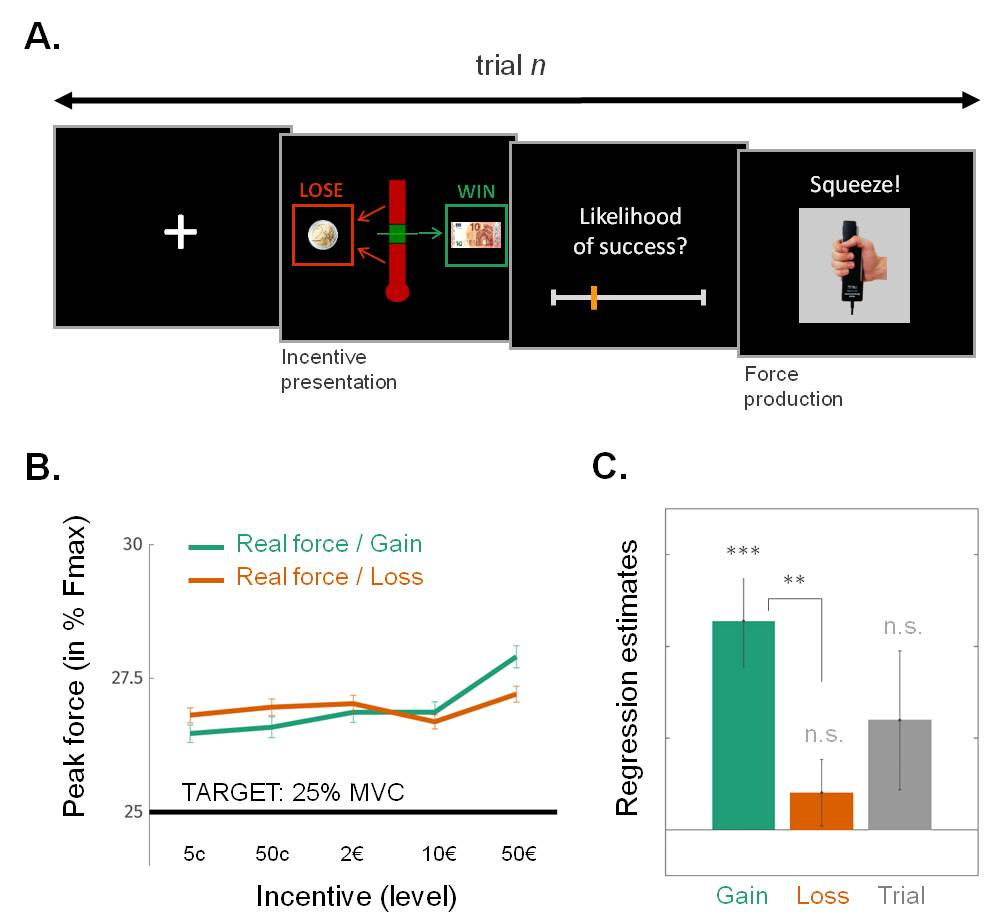
**

**Sup Figure 2. Influence of gain and loss prospects on force precision (Exp 5)**

1. Example trial. Participants (n=21) performed another variant of task design, similar to that used in Exp 4. Critical difference were 1) the absence of the quiz task, 2) the introduction of a confidence judgment on a rating scale prior to effort exertion, which is the matter of another paper, 3) the presentation of incentives as unique coins and notes (instead of multiple 10-cent images)
2. and (C) Same legend as previous figure. As in Exp 4, participants overshot the target (t(20)=3.5; p=0.0022). A significant positive modulation of peak force was found for potential gain (t(20)=4.6; p<0.001), but not for potential loss (t(20)=1.1; p=0.27), trial index (t(20)=1.6; p=0.12) or target size (t(20)=0.53; p=0.60. Moreover, the direct comparison of reward vs. loss estimates was significant (t(60)=3.6; p=0.0018).
